# Naked mole-rats maintain healthy skeletal muscle and Complex IV mitochondrial enzyme function into old age

**DOI:** 10.18632/aging.101140

**Published:** 2016-12-19

**Authors:** Elizabeth A Stoll, Nevena Karapavlovic, Hannah Rosa, Michael Woodmass, Karolina Rygiel, Kathryn White, Douglass M Turnbull, Chris G Faulkes

**Affiliations:** ^1^ LLHW Centre for Ageing and Vitality, Newcastle University, Newcastle upon Tyne, NE2 4HH, UK; ^2^ Wellcome Trust Centre for Mitochondrial Research, Institute of Ageing and Health, Newcastle University, Newcastle upon Tyne, NE2 4HH, UK; ^3^ Institute for Neuroscience, Newcastle University, Newcastle upon Tyne, NE2 4HH, UK; ^4^ Undergraduate Programme in Biomedical Sciences, Newcastle University, Newcastle upon Tyne, NE2 4HH, UK; ^5^ Electron Microscopy Research Services, The Medical School, Newcastle University, Newcastle upon Tyne, NE2 4HH, UK; ^6^ School of Biological & Chemical Sciences, Queen Mary University of London, London, E1 4NS, UK

**Keywords:** naked mole-rat, mitochondria, oxidative stress, animal models of aging, sarcopenia

## Abstract

The naked mole-rat (NMR) Heterocephalus glaber is an exceptionally long-lived rodent, living up to 32 years in captivity. This extended lifespan is accompanied by a phenotype of negligible senescence, a phenomenon of very slow changes in the expected physiological characteristics with age. One of the many consequences of normal aging in mammals is the devastating and progressive loss of skeletal muscle, termed sarcopenia, caused in part by respiratory enzyme dysfunction within the mitochondria of skeletal muscle fibers. Here we report that NMRs avoid sarcopenia for decades. Muscle fiber integrity and mitochondrial ultrastructure are largely maintained in aged animals. While mitochondrial Complex IV expression and activity remains stable, Complex I expression is significantly decreased. We show that aged naked mole-rat skeletal muscle tissue contains some mitochondrial DNA rearrangements, although the common mitochondrial DNA deletions associated with aging in human and other rodent skeletal muscles are not present. Interestingly, NMR skeletal muscle fibers demonstrate a significant increase in mitochondrial DNA copy number. These results have intriguing implications for the role of mitochondria in aging, suggesting Complex IV, but not Complex I, function is maintained in the long-lived naked mole rat, where sarcopenia is avoided and healthy muscle function is maintained for decades.

## INTRODUCTION

*Heterocephalus glaber*, the naked mole-rat (NMR), is a unique and fascinating mammal that has excited biologists since the discovery of its “insect-like” eusocial behavior [1]. While these animals have many unusual adaptations to their subterranean lifestyle, the discovery of their apparent resistance to cancer and exceptional longevity for a small rodent (over 30 years in captivity) has opened up new and important avenues of research [2]. The NMR is thus emerging as an exciting animal model for the study of aging.

These unique mammals exhibit negligible senescence, a phenomenon of very slow changes in the expected physiological characteristics with age, such as increased mortality and decreased physiological capacity [3]. One of the many consequences of normal aging in mammals is the devastating and progressive loss of skeletal muscle, termed sarcopenia [4]. While recent studies have reported that NMRs maintain vascular elasticity, cardiac function, gastrointestinal function, glucose tolerance, and reproductive capacity well into the third decade of life [5-7], no thorough investigation has been undertaken regarding the status of skeletal muscle tissue in the aging naked mole-rat.

Aging is thought to be caused in part by an accumulation of oxidative damage over time, which can lead to dysfunction of mitochondria. Found in all nucleated animal cells, mitochondria are subcellular organelles responsible for producing the majority of a typical cell's ATP demand [8] through oxidative phosphorylation (OXPHOS) at the inner mitochondrial membrane. Within a cell, mitochondria are the only extra-chromosomal source of DNA [9]; the circular, double-stranded, separately-replicating mitochondrial genome [10] spans 16,569 base pairs (bp) in humans and 16,386 bp in NMR. This genome contains only 37 genes [11], comprising less than 0.1% of genetic material that is exclusively maternally inherited. The previously published NMR mitochondrial genome [12] has high structural homology with the human equivalent, containing the same genes appearing in an identical order (Supp. Table 1).

Respiratory enzyme function is progressively lost in many tissues during normal aging both in humans [13] and in mice [14]. These defects are caused by deletions and point mutations in mitochondrial DNA (mtDNA) [13, 15]; the proximity of the mtDNA to OXPHOS activity and the relative lack of mtDNA repair systems [16] are believed to be responsible for its significantly higher mutation rate when compared to nuclear DNA [17]. Levels of damage to mtDNA, but not nuclear DNA, are inversely correlated with maximum lifespan in mammals [18]. In addition, mice genetically engineered to accumulate mtDNA mutations, due to defective DNA polymerase gamma, develop mito-chondrial dysfunction and a premature aging phenotype, including sarcopenia [19, 20]. Skeletal muscle tissue has high energy requirements and is subsequently greatly dependent on mitochondrial energy production. Thus skeletal muscle tissues suffer significantly from age-related accumulation of mtDNA deletions that result in mitochondrial dysfunction [13, 21].

It has been postulated that NMRs somehow avoid oxidative damage to attain longevity. Yet recent findings demonstrated that naked mole-rats have neither lower oxidative stress [22] nor superior anti-oxidant defence levels [23]. Indeed, a recent study showed that NMRs have significant oxidative damage to lipids, proteins, and DNA by two years of age [24]. These reports concluded that NMRs achieve longevity despite extensive oxidative damage. Most mammals, including mice and humans, accumulate mtDNA mutations and mitochondrial dysfunction with age, thought to be caused in part by oxidative stress. Here we assess whether such mitochondrial dysfunction is present in aging naked mole-rats.

In this study, we report that NMRs show no signs of age-related muscle atrophy into old age. Further, we demonstrate that Complex IV mitochondrial enzyme expression and function is largely maintained in the skeletal muscle tissues of aged animals. Meanwhile Complex I expression is significantly lower in aged animals, perhaps due to their high levels of oxidative stress. While NMRs do accumulate some mtDNA rearrangements, we find no evidence for common deletions observed in other mammals. Finally, we observe a significant increase in mitochondrial DNA copy number, whereas other mammals exhibit lower mitochondrial DNA copy number during normal aging. The maintenance of Complex IV enzymatic function may thus contribute to remarkably healthy aging in this mammal.

## RESULTS

### Integrity of skeletal muscle in aging naked mole-rats

**Figure 1:**
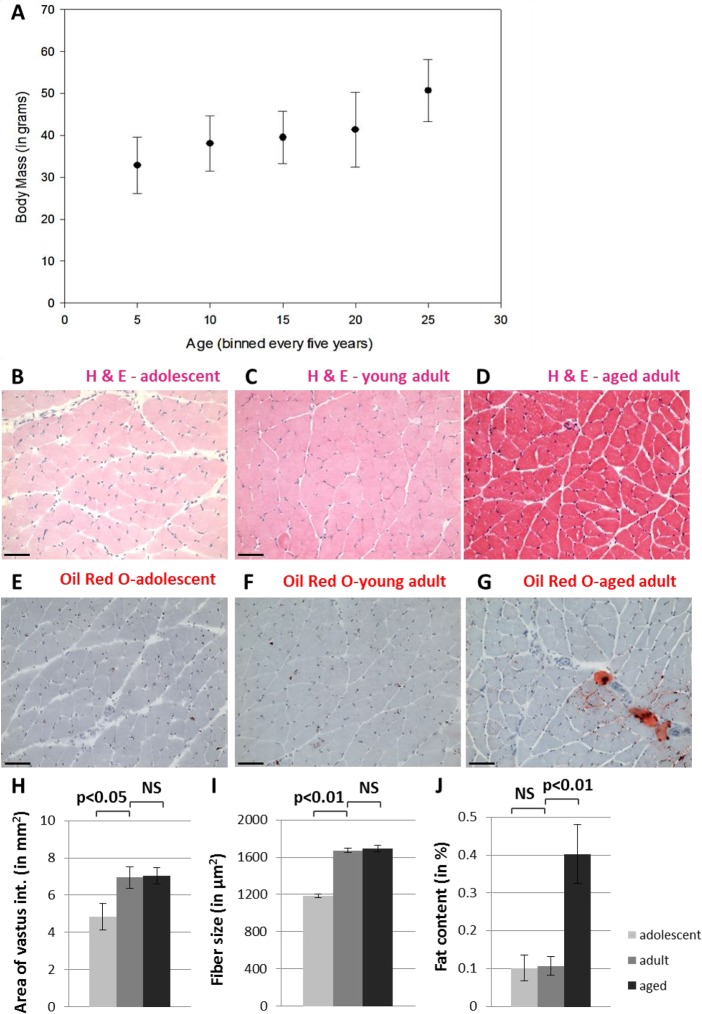
Naked mole-rats do not undergo muscle atrophy during the first twenty years of life NMR body size continues to grow into late middle age (**A**). Hematoxylin and eosin staining shows grossly normal quadriceps skeletal muscle in adolescent (**B**), young adult (**C**), and aged adult (**D**) NMR. Tissues from adolescent (**E**), young adult (**F**), and aged adult (**G**) NMR quadriceps muscle tissues stained with Oil Red O demonstrate fat deposits. Cross-sectional area of the entire vastus intermedius muscle increases significantly between adolescents and young adults, but does not change upon further aging (**H**). Individual skeletal muscle fibers grow significantly larger during maturation but do not change upon further aging (**I**). Fat content of the entire vastus intermedius muscle increases significantly during late adulthood (**J**). Scale bars signify 100 microns.

We conducted a comprehensive histological analysis of skeletal muscle tissue, specifically the vastus intermedius muscle of quadriceps, from adolescent (8 - 12 month old), young adult (4.5 - 6 year old), and aged adult (17.5 – 22 year old) NMRs (Figure 1). NMR skeletal muscle tissue stained with hematoxylin and eosin (Figure 1B-D) does not display pathological abnormalities such as angular fiber shape, non-peripheral nuclei, excess fibrous tissue or the presence of inflammatory cells, although aged muscle contains increased fat deposits (Figure 1D,G). Additional histological analysis demonstrates that the total cross-sectional area of this muscle and the size of individual muscle fibers (Figure 1H-I) increase during maturation (p<0.05 and p<0.001, respectively), but do not change size upon further aging (p>0.05, two-tailed t-test). The fat content within this muscle is significantly increased in aged adults compared with adolescents and young adults (Figure 1J, p<0.01). While muscle wasting causes loss of bulk in many mammals during aging, we find that NMRs do not lose body mass in the first twenty years of life (unpublished observations). These data, which support the maintenance of skeletal muscle integrity into old age, are broadly in agreement with reports from other laboratories who have observed age-related muscle atrophy in NMRs only above 28 years of age [25].

**Figure 2:**
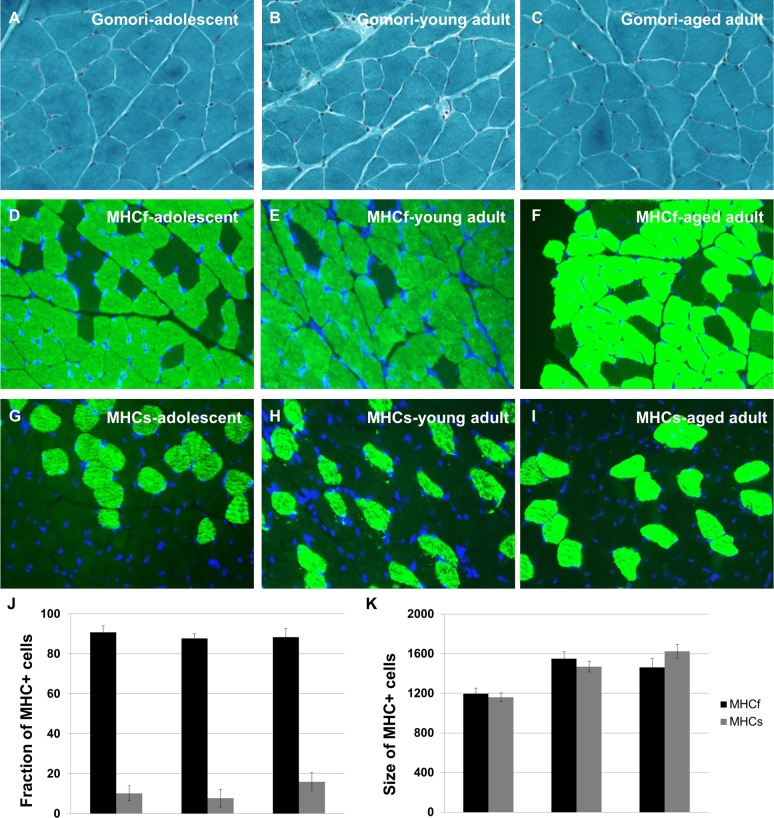
Naked mole-rats do not undergo changes in skeletal muscle integrity during aging Quadriceps muscle tissue sections were subjected to Gomori Trichrome Staining to reveal ragged red fibers; no such fibers were observed at any age (**A**-**C**). Quadriceps muscle tissue sections were immunohistochemically labelled with myosin heavy chain-specific antibodies to assess the relative numbers of fast-twitch and slow-twitch fiber types across the lifespan (**D**-**I**). No change in number was observed for either fiber type with age (**J**-**K**). Approximately 10% of muscle fibers in the naked mole-rat are MHCs+ at all ages studied, similar to the rat.

Gomori Trichrome Staining shows no incidence of ragged red fibers, signifying muscle fiber dysfunction, at any age (Figure 2A-C). In addition, we labelled tissue sections with myosin heavy chain-specific antibodies to assess the relative numbers of fast-twitch and slow-twitch fiber types across the lifespan, as these patterns have been shown to change with age (Figure 2D-I).

These data demonstrate that fast-twitch and slow-twitch fiber types do not change in number and do not show abnormal clumping patterns with age (Figure 2J-K). We find that 10% of muscle fibers in the naked mole-rat are MHCs+ at all ages studied, similar to the rat. These data support the lack of a sarcopenia phenotype in aging naked mole-rats.

### Mitochondrial function in aging naked mole-rats

**Figure 3:**
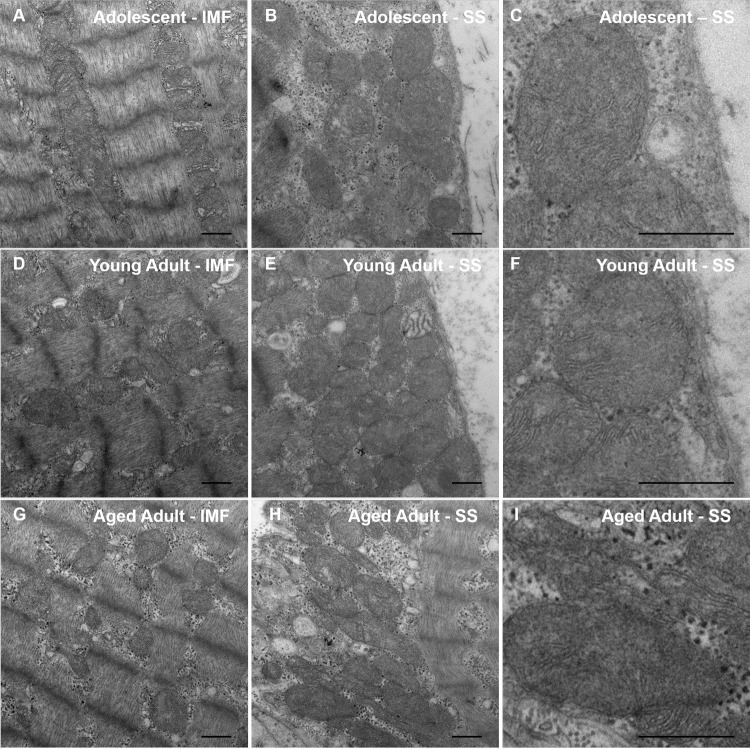
Transmission Electron Microscopy (TEM) of naked mole-rat skeletal muscle Naked mole-rat skeletal muscle tissue was fixed in glutaraldehyde and sectioned in preparation for TEM. Samples from each age group (adolescent, **A**-**B**; young adult, **C**-**D**; aged adult, **E**-**F**) were viewed in a blinded fashion. No obvious differences in ultrastructure are observed between samples. Specifically, no signs of age-related pathology such as autophagy or abnormal mitochondrial morphology are observed in any sample. Both intramyofibrillar (IMF) and subsarcolemmal (SS) regions are shown. Scale bars signify 500 nm.

To assess the ultrastructural integrity of skeletal muscle fibers in the aging naked mole-rat, we evaluated this tissue using transmission electron microscopy (Figure 3). No obvious differences were observed between the samples. In particular, no signs of age-related pathology such as autophagy or abnormal mitochondrial morphology were observed in any sample.

**Figure 4:**
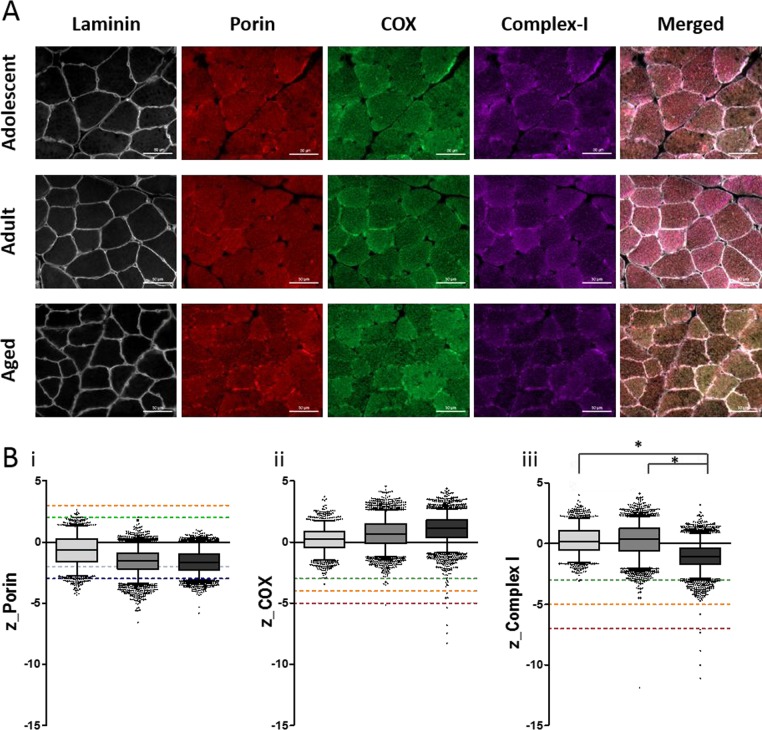
Quantitative quadruple immunofluorescence analysis reveals age-associated decreases in Complex I expression and no significant change in Complex IV expression (**A**) 10μm sections NMR skeletal muscle (quadriceps) stained for (left to right) laminin, VDAC (porin), MTCOI (COX) and NDUFB8 (complex-I), scale bar 50μm. (**B**) Boxplots for NMR grouped as adolescent (light grey, n=4), adult (middle grey, n=7) or aged (dark grey, n=4), where boxes represent the first quartile, median and third quartile, and whiskers show the 5^th^ and 95^th^ percentiles of z-scores for (i) porin (z<-3=very low, below dark blue dotted line; below dark blue dotted line;3<z<-2=low, below light blue dotted line; below dark blue dotted line;2<z<2=normal, below green dotted line; 2<z<3=high, below orange dotted line; z>3=very high, above orange dotted line), (ii) COX (z<-5=COX-negative, below red dotted line; below dark blue dotted line;5<z<-4=COX intermediate-negative, below orange dotted line; below dark blue dotted line;4<z<-3=COX intermediate positive, below green dotted line; z>-3= COX-positive, above green dotted line), and (iii) complex-I (z<-7=CI-negative, below red dotted line; below dark blue dotted line;7<z<-5=CI intermediate-negative, below orange dotted line; below dark blue dotted line;5<z<-3=CI intermediate positive, below green dotted line; z>-3= CI-positive, above green dotted line). Kruskal-Wallis test shows significant difference in complex-I levels between the three groups (p=0.023). A Mann-Whitney test confirms significant decrease in complex-I levels from adolescent to aged group (p=0.034) and adult to aged group (p=0.0182), shown by *.

We next quantified the mitochondrial enzyme expression by multiplexed immunofluorescence. 10μm sections of NMR skeletal muscle were stained with mitochondrial-encoded protein Complex IV (Subunit 1), Complex I (subunit 20), and nuclear-encoded mitochondrial protein Porin (Figure 4). This analysis identified age-associated decreases in Complex I expression, but no significant changes in Complex IV or Porin expression.

**Figure 5:**
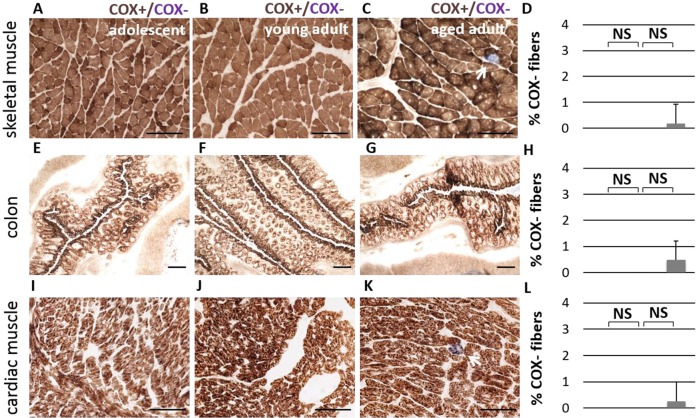
No significant difference in Complex IV activity is observed during aging Cytochrome *c* Oxidase activity (COX, in brown) is observed consistently in adolescent (**A**), young adult (**B**), and aged adult (**C**) NMR skeletal muscle tissue (quadriceps). A small number of COX-deficient cells (in blue) are observed in aged adults; this number does not significantly increase with age (**D**, p > 0.05, two-tailed t-test). Similar patterns were observed in colon tissue (**E**-**H**) and cardiac muscle (**I**-**L**).

To further investigate mitochondrial function during normal aging in the naked mole-rat, we first measured OXPHOS enzyme activity with combined histochemistry. Complex IV (cytochrome c oxidase, or COX) is an enzyme with both nuclear- and mito-chondrial-encoded subunits, which becomes dysfunctional when mtDNA is mutated, while succinate dehydrogenase (SDH) is a nuclear-encoded OXPHOS enzyme whose function is independent of mtDNA. Skeletal muscle fibers with functional COX activity are made visible by sequential oxidation-reduction steps in the presence of a catalyst. Cells with functional cytochrome c oxidase stain brown, while cells with insufficient COX enzyme activity stain only with SDH and appear blue. All fibers in the skeletal muscle tissue of adolescent and young adult NMRs stain COX+ (Figure 5A-B). By comparison, a very small number of COX-deficient cells were observed in aged adult animals (Figure 5C, p > 0.05, not significant). COX-deficient cells are very rare, comprising approximately 0.1% of total cells in older individuals (Figure 5D). By comparison, chronologically-matched (55-80 year old) humans and (3 year old) rats without skeletal muscle pathology exhibit COX-deficiency in approximately 2% of muscle fibers [26-28]. Other naked mole-rat tissues, specifically heart and colon, show little to no incidence of COX-deficient fibers with age (Figure 5).

### Rearrangements in the mitochondrial genome of aging naked mole-rats

**Figure 6:**
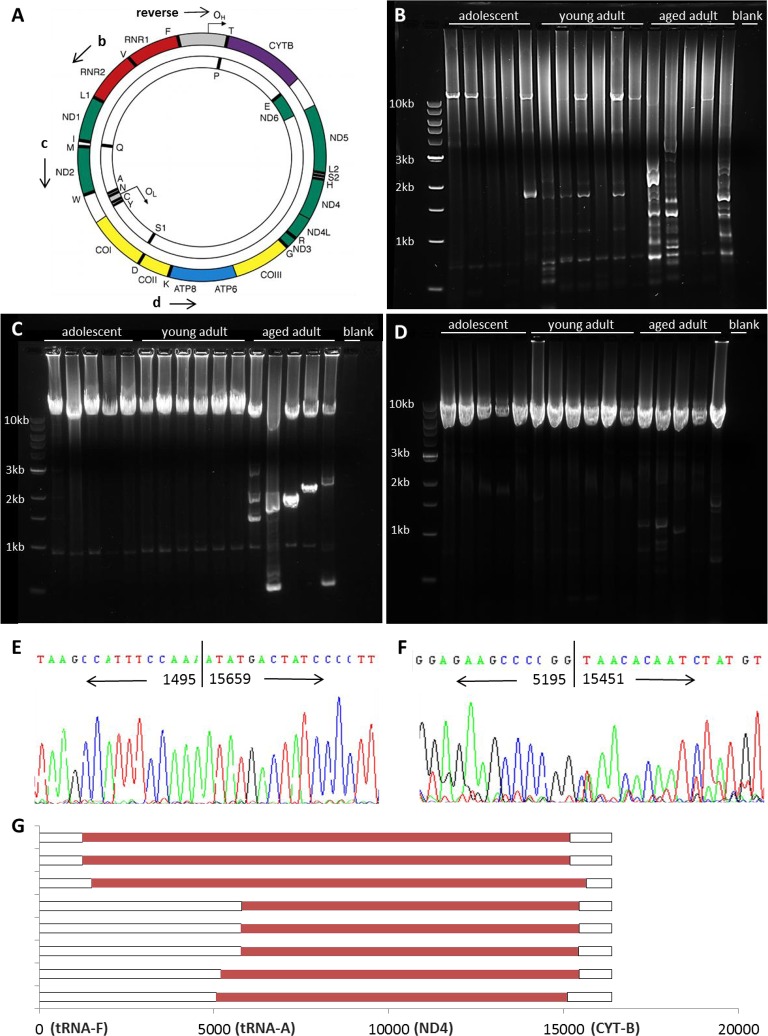
mtDNA rearrangements accumulate with age Shown is a schematic depicting the mitochondrial genome (**A**, adapted from [9]). Long-range PCR was used to amplify mtDNA, with a reverse primer at the origin of heavy strand replication (O_H_) within the displacement loop (DLOOP, in grey). The mitochondrial genome was amplified from RNR2 to DLOOP (14.7kb, **B**), from ND2 to DLOOP (11.6kb, **C**), and from ATP8 to DLOOP (8.1kb, **D**) to identify wild-type and altered mtDNA in homogenized quadriceps muscle. Shorter bands from young adult (**E**) and aged adult (**F**) samples were sequenced to identify the location of breakpoints in the NMR mitochondrial genome. Mapping of deletions is shown in red (**G**). 3′ breakpoints occur from 15110-15670 and 5′ breakpoints occur from either 1232-1495 or 5058-5803.

To test whether naked mole-rats accumulate deletions in mtDNA with age, we employed PCR-based amplifi-cation of the mitochondrial genome to search for wild-type (undamaged) and shortened (altered) copies of mtDNA (Figure 6A). Deletions in aging humans primarily occur in the major arc of the genome, between the origin of light strand replication (O_L_) and the origin of heavy strand replication (O_H_) which is located within the displacement loop (DLOOP, shown in gray) [29]. A variety of rearrangements are observed in aging human tissues, particularly the 4977bp common deletion, which renders a ∼11kb band in the entire linearized mitochondrial genome following long-range PCR and subsequent gel electrophoresis. In addition, very small bands (<3kb) are sometimes observed which are actually partial duplications of the mitochondrial genome rather than large-scale deletions [30]. NMR skeletal muscle samples were subjected to sequential rounds of long-range PCR to amplify virtually the entire genome, from tRNA-F to DLOOP (Figure 6B). Forward primers were then nested further into the genome to amplify smaller products concentrated on the major arc (Figure 6C-D). The differently-sized bands in each lane of a gel reflect the presence of wild-type and altered copies of mtDNA, allowing the identification of a molecular genetic signature in the skeletal muscle tissue of sixteen individual animals in three different age groups.

Adolescent NMRs have largely wild-type mtDNA, and the prevalence of shortened bands increases with age.

Wild-type mtDNA was amplified in most samples, although shorter templates amplify more readily, so less wild-type mtDNA may be observed in samples with a variety of deletions [31]. The locations of the breakpoints were narrowed down by nesting primers further into the genome (Figure 6C-D). We sequenced bands to verify the authenticity of shortened mtDNA fragments and to identify the precise location of breakpoints in the mitochondrial genome of young and aged adult NMRs, where the sequence jumps to one location to another. (Figure 6E-G). These results demonstrate the accumulation of what appear to be large deletions affecting the entire major arc of the NMR mitochondrial genome. Similar large deletions have been noted in human tissues and shown instead to be partial duplications of the minor arc [30]. It is notable that all rearrangements in naked mole-rat muscle tissues appear to be at least 9600bp in size; bands signifying smaller (true) deletions, such as the common deletion observed in human tissues, are not detected.

### Mitochondrial DNA copy number in aging naked mole-rats

**Figure 7:**
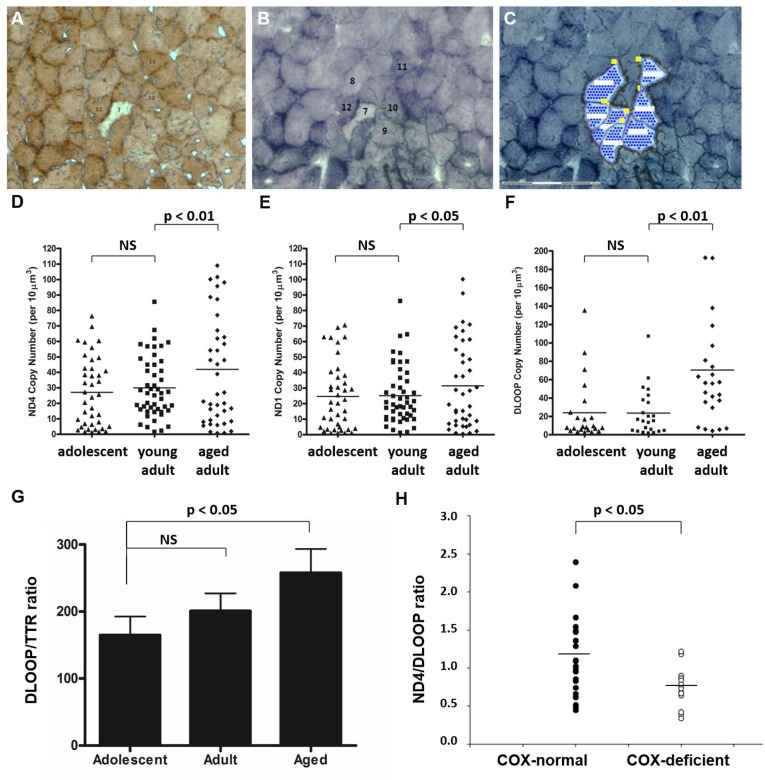
Total mtDNA copy number is significantly increased in aged skeletal muscle fibers NMR quadriceps muscle fibers with normal enzyme activity (such as Cells #11 and #12) were identified in COX-stained sections (**A**), correlated with SDH-stained sections (**B**), and laser-capture micro-dissected (**C**). mtDNA copy number was quantified by real-time PCR-based amplification of various regions of the mitochondrial genome. Total copy number in these cells, normalized to cell area, increased significantly in aged animals compared with young adult animals in all three regions quantified: ND4 (**D**, p<0.01), ND1 (**E**, p<0.05), and DLOOP (**F**, p<0.01). Homogenate tissues were subjected to real time PCR to compare the copy number of DLOOP with that of a single-copy nuclear gene, transthyretin (TTR). This normalized index, which gives the number of mitochondrial DNA copies per nucleus, also significantly increases with age (**G**). The few COX-deficient cells observed in aged NMR skeletal muscle (such as Cell #7) were laser-capture micro-dissected from SDH-stained serial sections and subjected to multiplexed real-time PCR to quantify the ratio of ND4 (a commonly deleted region) with DLOOP (the control region). COX-deficient cells from aged quadriceps muscle have a significantly lower ND4/DLOOP index than normal cells in aged quadriceps muscle (**H**).

To quantify mtDNA copy number at various loci, we first collected individual muscle fibers by laser-capture micro-dissection (Figure 7A-C). We subsequently subjected samples to real-time PCR to measure total mtDNA copy number, after optimizing newly customized real-time primer-probe sets for this species (Supp. Table 4). mtDNA copy number increases significantly in aged animals compared with adolescent and young adult animals in a number of regions of the mitochondrial genome, according to measures of ND4 (Figure 7D, p<0.01), ND1 (Figure 7E, p<0.05), and DLOOP (Figure 7F, p<0.01). This up-regulation of copy number may provide sufficient wild-type mtDNA to maintain mitochondrial function in later life despite rearrangements in the mitochondrial genome. The few COX-deficient cells observed in aged NMR skeletal muscle were also laser-capture micro-dissected from SDH-stained serial sections and subjected to multiplexed real-time PCR to quantify the ratio of ND4 (a commonly deleted region) with DLOOP (the control region). COX-deficient cells from aged quadriceps muscle have a significantly lower ND4/DLOOP index than normal cells in aged quadriceps muscle, indicating mtDNA deletions in this small cell population (Figure 7H).

## DISCUSSION

In this report we demonstrate that NMRs show few signs of age-related muscle atrophy or mitochondrial dysfunction into old age. We show that healthy skeletal muscle tissue is maintained for decades in the naked mole-rat (Figures 1-2). We find that skeletal muscle health is associated with normal mitochondrial morphology (Figure 3), unchanged expression levels of Complex IV and decreased expression levels of Complex I (Figure 4) and a virtual lack of Complex IV-deficient fibers (Figure 5). mtDNA from naked mole-rat skeletal muscle shows some rearrangement in long-range PCR assays (Figure 6), but the common deletions observed in other species such as mouse and human are not observed in the naked mole-rat. Finally, we conclude that increases in overall mtDNA content (Figure 7) may support continued mitochondrial enzyme function despite some genomic rearrangements.

In this study we observe age-related increases in mtDNA copy number in NMR tissues. Most mammals, including humans, exhibit an age-related *decrease* in mtDNA copy number in skeletal muscle [13] and other tissues [32]. Interestingly, however, age-associated *increases* in mtDNA copy number have been observed in mice which nurse a lifelong mutation burden due to possessing a proofreading-deficient form of mitochondrial DNA polymerase gamma [33]. Perhaps NMRs demonstrate a similar response to their high levels of oxidative stress, which constitutively cause oxidative lesions to DNA, proteins, and lipids from an early age [24].

Early theories of aging postulated that oxidative stress was associated with negative health. Yet there is little evidence to support a connection between oxidative stress and age-related mtDNA deletions. Naked mole-rats display both high levels of oxidative stress and extreme longevity [22]. Studies in this intriguing species do not support a connection between oxidative stress and longevity, or oxidative stress and mitochondrial dysfunction.

However, healthy aging and the maintenance of mito-chondrial function may indeed be intimately connected. While a great deal of literature demonstrates that mitochondrial dysfunction is normally associated with age-related deficits, including sarcopenia, our findings demonstrate the reverse is also true: successful aging is associated with the overall maintenance of mitochondrial health. Previous studies have demonstrated loss of both Complex IV and Complex I in sarcopenic skeletal muscle in aged rodents, however it is unclear whether these changes are causative in the aging process [28, 34-36]. Indeed Complexes I and IV have rarely been studied in individual myofibers at protein level or transcript level so the effects at a cellular level are not well-understood. Furthermore, causal mechanism is extraordinarily difficult to parse as genetic techniques such as knockout mice cannot target mitochondrial DNA.

The results in this report demonstrate that mitochondrial integrity and sustained mitochondrial Complex IV enzyme activity are associated with healthy aging, even in the presence of lower Complex I expression, in the naked mole-rat. Given that Complex IV function has been tied to loss of skeletal muscle function during normal ageing, it is intriguing that Complex IV enzyme activity is maintained in a long-lived and healthy mammal that does not show age-related sarcopenia.

## METHODS

### Animals

Naked mole-rats (NMRs) were raised in compliance with institutional guidelines, in colonies maintained by Dr Chris Faulkes at Queen Mary University of London. All animals were born in captivity and kept under constant ambient temperature of 28-30°C. Animals were housed in artificial burrow systems composed of interconnected perspex tubing with separate chambers for nesting and a latrine, simulating burrow conditions of their natural habitat. They were fed an ad libitum diet of a variety of chopped root vegetables such as sweet potato and turnip. There was no set lighting regime within the room as naked mole-rats are effectively blind, and the enclosed environment of the artificial burrow system maintained the necessary high humidity preferred by the animals.

Skeletal muscle tissues were collected from 16 healthy male naked mole rats: five 8-12 month old NMRs, six 4.5-6 year old NMRs, and five 17-22 year old NMRs. Animals were anesthetized with CO_2_ and euthanized by cervical dislocation. Blood was flushed out by transcardiac perfusion of saline. Tissues including quadriceps muscle were collected and flash frozen in super-cooled isopentane prior to storage at −80°C. 10μm serial transversely orientated slices of quadriceps muscle were obtained using a cryostat (Microm HM560, Thermo Fisher) for histological analysis of skeletal muscle tissue.

### Histological analysis of skeletal muscle integrity

Tissue sections were subjected to Oil Red O staining to demonstrate fat within skeletal muscle. Sections on glass slides were fixed for 1 hour in Formal Calcium (100mL of 37% Formalin, 900mL of water, 4.4g CaCl_2_), then washed three times in deionized water (dH_2_O). Oil Red O (0.5g Oil Red O, 500mL isopropanol) was diluted immediately before use as follows: 45mL stock solution in 30mL dH_2_O. Sections were then incubated at room temperature with diluted Oil Red O, then washed three times with dH_2_O. Sections were stained for 10 minutes in Meyer's Hematoxylin to demonstrate cell nuclei, then washed in tap water. Coverslips were mounted with Citifluor mounting medium. Sections were analyzed using StereoInvestigator software, receiving input from an Olympus BX51 microscope. The diameter of each section was quantified across four locations to calculate total area of the round vastus intermedius muscle. Fiber size (area) was quantified in 100 cells stereologically sampled across tissue sections from each NMR. The percentage of fat area (of total vastus intermedius area) was quantified in four sections for each NMR. Results were compared between groups using paired t-tests in Excel.

Sections were subjected to Hematoxylin & Eosin staining to demonstrate age-associated pathology in skeletal muscle tissue. Sections on glass slides were fixed for 5 minutes in Formal Calcium (100mL of 37% Formalin, 900mL of water, 4.4g CaCl_2_), then washed in tap water for 5 minutes. Sections were placed in Meyer's Hematoxylin to stain for nuclei for 3 minutes, then washed in tap water. Sections were then placed in Eosin to stain muscle fibers for 3 minutes, and subsequently washed in tap water. Sections were then dehydrated in ascending alcohol solutions (70% ethanol for 1 × 5 min, 95% ethanol for 1 × 5 min, 100% ethanol for 2 × 5 min, followed by Histoclear HS-200 (2 × 10 min)). Coverslips were mounted with DPX. Sections were observed at 20x to assess gross fiber shape, location of nuclei, presence of adipose tissue fibrous tissue, and mononucleated inflammatory cells.

Sections of quadriceps muscle were subjected to Gomori Trichrome staining to demonstrate histopathological abnormalities in skeletal muscle such as ragged red fibers. To make the solution, 0.6g of phosphotungstic acid was added to 0.6mL acetic acid in 200mL of deionized water (dH_2_O), and the pH was adjusted to 3.4 using NaOH. This solution was divided into two 100mL solutions. 1.2g Chromotrope 2R was added to create a 100mL red solution and 0.3g Fast Green FCF was added to create a 100mL turquoise solution. These two solutions were stored at room temperature and mixed together immediately before use to make Gomori Trichrome stain. Fresh frozen sections were immersed in Meyer's Hematoxylin for 10 minutes, then rinsed with tap water until clear. Sections were then immersed in Gomori Trichrome for 10 minutes, and subsequently differentiated with 0.2% acetic acid until cleared. Sections were then immediately immersed in 70% ethanol, and dehydrated in ascending alcohol solutions (95% ethanol for 1 × 5 min, 100% ethanol for 2 × 5 min, followed by Histoclear HS-200 (2 × 10 min)). Coverslips were mounted with DPX. Sections were observed at 20x to determine the presence of histopathology. Nuclei appeared purple and normal muscle myofibrils appeared turquoise.

Sections of quadriceps muscle were subjected to immunohistochemical staining to assess skeletal muscle fiber subtypes. Slides were post-fixed in 4% cold paraformaldehyde, then rinsed with PBS. Sections were incubated for one hour at room temperature in blocking solution (5% donkey serum and 0.1% Triton-X in PBS), then incubated overnight at 4°C with primary mouse monoclonal antibody diluted in blocking solution (antibody details in Supp. Table 2). Sections were rinsed with PBST (0.1% Triton-X in PBS), incubated in donkey anti-mouse secondary antibody, rinsed with PBST, and co-labeled with Hoechst nuclear dye (1μg/mL). Coverslips were mounted with Citifluor. Sections were observed using a Zeiss AxioImager. The fraction of slow-twitch and fast-twitch skeletal muscle fibers was compared between age groups using a two-tailed t-test in Excel.

### Transmission electron microscopy

To assess ultrastructure of intramyofibrillar and subsarcolemmal mitochondria, skeletal muscle tissue was subjected to transmission electron microscopy (TEM). Sections were fixed in 2% glutaraldehyde in 0.1M cacodylate buffer, post-fixed in 1% osmium tetroxide, dehydrated in acetone and embedded in epoxy resin. Ultrathin sections were taken both longitudinally and transversely through the tissue blocks, stained with uranyl acetate and lead citrate and examined on a Philips CM100 TEM.

### Histological analysis of mitochondrial enzymes

10μm thick sections of transversely orientated skeletal muscle (quadriceps) were cut for 15 animals (one animal was excluded due to insufficient tissue). Two sections from each animal were required for this protocol; one designated OXPHOS and one as a no primary control (NPC). Sections were left to air dry for one hour prior to fixing in cold 4% paraformaldehyde, washing in 0.25% TBS-T (0.25% Tween-20 in TBS) and permeabilisation along a methanol gradient. Following blocking in 10% normal goat serum (Sigma), OXPHOS sections were coated with a cocktail of primary antibodies, while NPC sections were stained only with laminin antibody. Slides were incubated at 4°C overnight in a humidified chamber then washed with 0.25% TBS-T. Both OXPHOS and NPC slides were incubated with secondary antibody cocktail at 4°C for 2 hours. After further washing with 0.25% TBS-T, all slides were incubated with a streptavidin-conjugated antibody for 2 hours at 4°C. Slides were then washed for the final time with 0.25% TBS-T and mounted in Prolong Gold (Sigma).

Sections were imaged at 20x magnification using a Zeiss Axioimager M1 with a motorised stage and ZEN 2011 (blue edition) to create tiled, mosaic images of the entire biopsy section. Exposure times were optimised for each channel to avoid saturation. Image analysis was performed in Imaris software (bitplane), using laminin staining to mask the borders of the muscle fibers and create surfaces to measure the immuno-reactivity of COXI, NDUFB8 and porin in each muscle fiber as outlined in [37].

Fresh frozen tissue sections were subjected to a histochemical assay to demonstrate mitochondrial enzyme activity. Cells with functional enzyme activity are made visible by sequential oxidation-reduction steps in the presence of a catalyst. This combined enzymatic assay provides Cytochrome c Oxidase (COX) with the electron donor 3-3′-diaminobenzidine (DAB), which forms an insoluble brown reaction product in the presence of functional enzyme. The SDH incubation medium uses Nitro Blue Tetrazolium (NBT) as an electron acceptor; when reduced, NBT forms an insoluble microcrystalline blue formazan. Cells are immersed in cytochrome c oxidase (COX) incubation medium (20% cytochrome *c* substrate and 80% DAB, vortexed with catalase crystals), then succinate dehydrogenase (SDH) incubation medium (79% nitroblue tetrazolium, 10% sodium succinate, 10% phenazine methosulfate and 1% sodium azide). Sequential staining renders cells with COX activity brown; any cells with insufficient COX activity to block the DAB reaction subsequently turn blue. Since COX subunits are mitochondrially-encoded, deficiency of this enzyme is a marker of mtDNA mutations; meanwhile SDH is nuclear encoded, so the expression and activity of this enzyme is independent of mtDNA mutations [38]. COX-deficient (blue) cells were quantified as a percentage of total cells in stereologically-selected sample fields throughout the vastus intermedius muscle. The fraction of COX-deficient cells in skeletal muscle tissue was compared between age groups using a two-tailed t-test in Excel.

For the purpose of molecular analysis in later experiments, the sections were not sequentially stained with both media as DAB is known to inhibit PCR reactions [39]. In such cases, serial slices of quadriceps tissue were used for separate COX and SDH staining. The pairs of sample slides were placed in a 37°C incubator for 15 minutes before being washed with phosphate-buffered saline (PBS). COX staining was used to determine the location of desirable cells for laser-capture microdissection based on COX activity. Matched samples were then laser-captured from SDH-stained sections for downstream PCR applications (See Multiplexed real-time PCR).

### Analysis of wild-type and altered mtDNA by long-range PCR

The naked mole-rat mitochondrial genome was compared to the human equivalent using the NCBI database (GenBank NC_015112.1 and GenBank NC_012920.1 respectively) for existing genes as well as their relative order and size (Supp. Table 1). Primers were custom-designed based on the recently-sequenced NMR mitochondrial genome [12, 40], the known locations of human minor and major arcs [41], and the support of both Primer3 (http://primer3.wi.mit.edu/) and Primer-BLAST (http://www.ncbi.nlm.nih.gov/tools/primer-blast/) for the purpose of comparison and verification.

To amplify the majority of the 16.3kb NMR mitochondrial genome using two rounds of Long-Range PCR, the synthesis of nested primers is required [42]. The nesting shortens the PCR product with two sequential reactions. This process allows for replenishment of the PCR reagents and further amplification of the product, which helps to obtain a satisfactory signal that can be visualised with gel electrophoresis. Multiple forward and reverse primers were designed; shown in Supp. Table 3 are the locations and sequences of the primers which successfully amplified the expected 14.7kb, 11.6kb, and 8.1kb products from NMR skeletal muscle tissues homogenized from each animal.

The long-range PCR was executed in 25 μL reaction mixtures containing 2 μL of sample DNA, 14.25 μL dH_2_O, 0.4mM Forward Primer, 0.4mM Reverse Primer, 1X LA Buffer (Mg^2+^ plus), 0.4mM dNTP Mix and 0.25 μL LA-Tak polymerase from the LATak kit (TaKaRa BIO Inc.). DEPC dH_2_O was used as negative control. The following conditions were used for PCR reactions (Veriti 96 Well Thermal Cycler, Applied Biosystems): initial denaturation at 94°C for 1 minute, 30 cycles to amplify the product (94°C for 20 seconds, 60°C for 20 seconds, and 68°C for 1 minute per 1kb predicted product size), and a final extension of 68°C for 10 minutes.

2μl of the product obtained from First Round reaction was used as a template for the Second Round of PCR. The Second Round of PCR was essentially identical to the first except that stage 2 involved 20 cycles instead of 30 and nested primers were used (Supp. Table 3). For both Rounds, reaction mixtures were kept on ice and only placed into the Thermal Cycler once it had reached 94°C. 8 μL of Second Round amplified products were combined with 2 μL Loading buffer and separated using a GelRed Nucleic Acid (Biotium) stained 0.8% agarose gel, electrophoresed at 85V for 3 hours. The products were ran alongside a Quick-Load^®^ 1Kb DNA Ladder (New England BioLabs Inc.) and visualized for evaluation under UV light (BioRad ChemiDoc™ MP Imaging System).

### Sequencing of altered copies of mtDNA from long-range PCR products

Long-range PCR was performed as previously, and putative deletion bands were extracted from agarose gels and purified. 5 μL samples were then digested with 1.5 μL ExoI (Thermo-Fisher EN0582)/Shrimp Alkaline Phosphatase (Promega M9910) at 37°C for 15 minutes, followed by inactivation of the enzymes at 80°C for 15 minutes. Samples were cycle-sequenced to incorporate M13-tagged primers (Eurofins, 3.2μM concentration) using BigDye Reaction Buffer (ABI 402824) and BigDye v3.1 Kit (ABI 4337456). 13 μL of 1X BigDye MasterMix was added to samples, and these reactions were subject to the following conditions: denaturation at 96°C for 1 minute, then 25 cycles of 96C for 10s, 50°C for 5s, and 60°C for 4 minutes. Samples were pre-cipitated with EtOH, subjected to heat-shock in the presence of 10 μL Hi-Di Formamide, snap-frozen on ice, then sequenced using an ABI3130 Genetic Analyzer. Sequences were analyzed with SeqScape Software. 1/3 of deletion bands were sequenced.

### Single-cell laser-capture micro-dissection and DNA isolation

Three animals from each of the age groups were blindly selected. COX activity was assessed in neighbouring individual skeletal muscle fibers and the corresponding SDH-stained cells from serial sections were microdissected using the Zeiss PALM MicroBeam Laser-Capture Micro-dissection Microscope. The cells were selected based on the presence of at least one distinctly COX-deficient cell surrounded by gradient, intermediate and/or normal COX+ cells (example shown in Figure 7A-C). 10 μL of freshly prepared cell lysis buffer (50mM Tris-HCl pH8.5, 0.5% Tween–20 and 1% Proteinase K in 390 μL DEPC Treated Water) was placed in the caps of 200μl collection tubes. After catapulting and collection, tubes were centrifuged at 10,000 rpm for 10 minutes, then lysed for five hours at 55°C (Veriti 96 Well Thermal Cycler, Applied Biosystems). Proteinase K was heat inactivated at 95°C for 10 minutes and cell lysate were stored at −20°C until required for use in Real-Time PCR.

### mtDNA copy number and deletion level analysis using real-time PCR

Real-time PCR was used to analyse mtDNA copy number in single cells by quantifying several regions: ND1, ND4, and DLOOP. The real-time PCR was based on the procedure described previously [43] which quantifies fluorescence emitted by dyes VIC and FAM which are conjugated to probes complementary to regions of mtDNA. A 20 μL reaction volume contained 10 μL of TaqMan Universal Master Mix II with UNG (Applied Biosystems), 4 μL of DEPC dH_2_O and 1 μL each of the following: DLOOP forward primer, DLOOP reverse primer, DLOOP-FAM probe, or ND1 and ND4 forward primer, ND1 and ND4 reverse primer, ND1-VIC probe and ND4-FAM probe (Life Technologies). Details of the primers and probes for each assay are described in Supp. Table 4. 2 μL of sample DNA (laser-microdissected tissue) were run in triplicate, alongside 5 point 10-fold dilutions of an adolescent homogenate sample of known concentration and a blank (dH_2_O). The assay was performed on the StepOnePlus™ Real-Time PCR System (Applied Biosystems). The PCR reaction conditions used were: incubation at 50°C for 2 minutes followed by denaturation at 95°C for 10 minutes, after which amplification occurred over 40 cycles consisting of 15 seconds at 95°C and 1 minute at 60°C. Six samples from each NMR were each run in triplicate to quantify total copy number. Each sample was normalized to the standard curve, using the equation VALUE = (CT – INTERCEPT)/SLOPE. These values were normalized to total area of laser-captured samples. Total copy number of mtDNA in ND1, ND4, and DLOOP regions were compared between age groups using a two-tailed t-test in Excel and plotted in Prism.

## SUPPLEMENTARY METHODS AND TABLES


