# Supplementary material for: Naked mole-rats maintain healthy skeletal muscle and Complex IV mitochondrial enzyme function into old age
Source: Aging (Albany NY). 2016 Dec 19;8(12):3468–83. doi: 10.18632/aging.101140 (PMC5270680; doi:10.18632/aging.101140)
Supplement: Supplementary file 1 [file aging-08-3468-s001.pdf]

SUPPLEMENTARY MATERIAL

Supplementary Table 1. Comparison of human and naked mole rat mitochondrial genome. A table showing the genes present, their consecutive order and corresponding length of the human mitochondrial genome compared to the NMR equivalent. (L) denotes the gene is located on the light strand of genome. Areas in gray highlight genes that are different sizes in human and naked mole rat. Sequences were accessed and analysed in NCBI.

	Human mtDNA order:			Naked mole-rat mtDNA order:		
	Position	Length	Gene	Position	Length	Gene
MINOR ARC (uncommonly deleted)	577-647	71	tRNA-F	1-68	68	tRNA-F
	648-1601	954	RNR1	69-1035	967	12S rRNA
	1602-1670	69	tRNA-V	1035-1100	66	tRNA-V
	1671-3329	1,659	RNR2	1101-2651	1,551	16S rRNA
	3230-3304	75	tRNA-L	2652-2726	75	tRNA-L
	3307-4262	955	ND1	2731-3688	958	ND1
	4263-4331	69	tRNA-I	3689-3757	69	tRNA-I
	4329-4400 (L)	72	tRNA-Q	3755-3825 (L)	71	tRNA-Q
	4402-4469	68	tRNA-M	3828-3895	68	tRNA-M
	4470-5511	1,042	ND2	3896-4937	1,041	ND2
	5512-5579	68	tRNA-W	4938-5006	69	tRNA-W
	5587-5655 (L)	69	tRNA-A	5010-5078 (L)	69	tRNA-A
	5657-5729 (L)	72	tRNA-N	5079-5151 (L)	73	tRNA-N
MAJOR ARC (commonly deleted)	5761-5826 (L)	65	tRNA-C	5186-5251 (L)	66	tRNA-C
	5826-5891 (L)	65	tRNA-Y	5252-5318 (L)	67	tRNA-Y
	5904-7445 (L)	1,542	COX1/COI	5320-6861	1,541	COX1/COI
	7446-7514 (L)	68	tRNA-S1	6863-6931 (L)	69	tRNA-S1
	7518-7585	68	tRNA-D	6938-7006	69	tRNA-D
	7586 8269	683	COX2/COII	7007 7690	684	COX2/COII
	8295 8364	70	tRNA K	7693 7761	69	tRNA K
	8366-8572	207	ATP-8	7763-7966	204	ATP-8
	8527-9207	681	ATP-6	7924-8604	681	ATP-6
	9207-9990	784	COX3/COIII	8604-9387	784	COX3/COIII
	9991-10058	68	tRNA-G	9388-9455	68	tRNA-G
	10059-10404	346	ND3	9456-9801	345	ND3
	10405-10469	65	tRNA-R	9803-9873	71	tRNA-R
	10470-10766	297	ND4l	9875-10171	297	ND4l
	10760-12137	1,378	ND4	10165-11542	1,378	ND4
	12138-12206	69	tRNA-H	11543-11611	69	tRNA-H
	12207-12265	59	tRNA-S2	11612-11670	59	tRNA-S2
	12266-12336	71	tRNA-L2	11670-11739	70	tRNA-L2
	12337-14148	1,812	ND5	11740-13551	1,811	ND5
	14149-14673 (L)	525	ND6	13548-14078 (L)	531	ND6
	14674-14742 (L)	69	tRNA-E	14079-14146 (L)	68	tRNA-E
	14747-15887	1,141	CYTB	14149-15283	1,135	CYTB
	15888-15953	66	tRNA-T	15289-15357	69	tRNA-T
	15956-16023 (L)	68	tRNA-P	15360-15425 (L)	66	tRNA-P
D-loop	1-576 and 16024-16569	1,121	D loop	15426 16386	961	D-loop

Supplementary Table 2. Antibodies used for immunohistological analysis. Shown is a list of antibodies used in Figures 2 and 4 of this study.

Antigen	Source	Dilution
Complex I–subunit 20kDa NDUFB8 (mouse IgG1)	Abcam (Ab110242)	1:100
Complex IV–subunit 1 MTCO1 (mouse IgG2a)	Abcam (Ab14705)	1:100
Porin (mouse IgG2b)	Abcam (Ab14734)	1:50
Laminin (rabbit IgG)	Sigma-Aldrich (L9393)	1:50
Myosin Heavy Chain-Slow (mouse IgG)	Novacastra (NCL-MHCs)	1:200
Myosin Heavy Chain-Fast (mouse IgG)	Novacastra (NCL-MHCf)	1:200
Goat Anti-IgG1 biotin	Jackson IR Lab (115-065-205)	1:200
Goat Anti-IgG2a Alexa Fluor 488 nm	Life Technologies (A21131)	1:200
Goat Anti-IgG2b Alexa Fluor 546 nm	Life Technologies (A21143)	1:100
Goat Streptavidin Alexa Fluor 647 nm	Life Technologies (S31556)	1:100
Goat Anti-rabbit Alexa Fluor 405 nm	Life Technologies (A31556)	1:200
Donkey Anti-mouse Alexa Fluor 546 nm	Jackson IR Lab (711-295-152)	1:200

Supplementary Table 3. Primers used in long-range experiments shown in Figure 6. Shown are primers used for long-range PCR to amplify large parts of the mitochondrial genome for experiments shown in Figure 6.

Primer Region	Sense	Location	Sequence (5'→3')	Used in	
t-RNA-F	Forward	nt 131-150	CGC CAG TGA GAA TGC CCT TA	Round I	
D-Loop	Reverse	nt 15,881–15,862	CTG GAA GCA CCA AAC CAT CG	Round I	
RNR2 (16S)	Forward	nt 1,212-1,232	AGA GGT GAA AAG CCT ACC GA	Round II	14,669bp product
D-Loop	Reverse	nt 15,881–15,862	CTG GAA GCA CCA AAC CAT CG	Round II	
ND2 (I)	Forward	nt 4,039–4,058	AAG CCC ACG ATC CAC AGA AG	Round I	
t-RNA-F	Reverse	nt 150–131	TAA GGG CAT TCT CAC TGG CG	Round I	
ND2 (II)	Forward	nt 4,239–4,258	TGC CCG AAG TAA CTC AAG GG	Round II	11,642bp product
D-Loop	Reverse	nt 15,881–15,862	CTG GAA GCA CCA AAC CAT CG	Round II	
t-RNA-A	Forward	nt 5,039-5,058	ACT GGA CGC AAA CCA AAC AC	Round I	
D-Loop	Reverse	nt 15,881–15,862	CTG GAA GCA CCA AAC CAT CG	Round I	
ATP8	Forward	nt 7,765-7,786	GCC ACA ACT AGA CAC ATC AAC G	Round II	8,116bp product
D-Loop	Reverse	nt 15,881–15,862	CTG GAA GCA CCA AAC CAT CG	Round II	

Supplementary Table 4. Primers used in real-time PCR experiments shown in Figure 7. Shown are details of primers/probes used for real time PCR to quantify copy number, as shown in Figure 7. All primers and probes were designed based on the naked mole-rat genome sequence: http://www.ncbi.nlm.nih.gov/nuccore/NC_015112.1

Primer Region	Sense	Location	Sequence (5'→3')	Used in	
t-RNA-F	Forward	nt 131-150	CGC CAG TGA GAA TGC CCT TA	Round I	
D-Loop	Reverse	nt 15,881–15,862	CTG GAA GCA CCA AAC CAT CG	Round I	
RNR2 (16S)	Forward	nt 1,212-1,232	AGA GGT GAA AAG CCT ACC GA	Round II	14,669bp product
D-Loop	Reverse	nt 15,881–15,862	CTG GAA GCA CCA AAC CAT CG	Round II	
ND2 (I)	Forward	nt 4,039–4,058	AAG CCC ACG ATC CAC AGA AG	Round I	
t-RNA-F	Reverse	nt 150–131	TAA GGG CAT TCT CAC TGG CG	Round I	
ND2 (II)	Forward	nt 4,239–4,258	TGC CCG AAG TAA CTC AAG GG	Round II	11,642bp product
D-Loop	Reverse	nt 15,881–15,862	CTG GAA GCA CCA AAC CAT CG	Round II	
t-RNA-A	Forward	nt 5,039-5,058	ACT GGA CGC AAA CCA AAC AC	Round I	
D-Loop	Reverse	nt 15,881–15,862	CTG GAA GCA CCA AAC CAT CG	Round I	
ATP8	Forward	nt 7,765-7,786	GCC ACA ACT AGA CAC ATC AAC G	Round II	8,116bp product
D-Loop	Reverse	nt 15,881–15,862	CTG GAA GCA CCA AAC CAT CG	Round II	
